# Influence of ceramic thickness, background and cement shade on the translucency of zirconia reinforced lithium silicate and lithium disilicate ceramics

**DOI:** 10.4317/jced.60806

**Published:** 2023-09-01

**Authors:** Ozge Sancaktar, Merve Koseoglu, Funda Bayindir

**Affiliations:** 1 Graduate Prosthodontist, Private Practice, Istanbul, Turkey; 2 Associate Professor, Sakarya University, Faculty of Dentistry, Department of Prosthodontics, Sakarya, Turkey; 3 Professor, Ataturk University, Faculty of Dentistry, Department of Prosthodontics, Erzurum, Turkey

## Abstract

**Background:**

Although, the influence of cement and background shade on the final color and translucency of zirconia reinforced lithium silicate (ZLS) and lithium disilicate (LDS) ceramics has been previously investigated, there is still little data on the translucency of LDS and ZLS ceramics in decreased thickness (0.4 and 0.6 mm). The aim of this study was to investigate ceramic thickness’, background and cement shades’ effects on the zirconia reinforced lithium silicate (ZLS) and lithium disilicate (LDS) ceramics’ translucency.

**Material and Methods:**

Totally 160 square-shaped A1 shade LDS and ZLS samples produced in 0.4 and 0.6 mm thicknesses were cemented with light and neutral shade resin cement on A2 and A3 shade composite resin backgrounds. The color notations of specimen were measured and translucency parameter (TP) values were calculated. Factorial ANOVA and Tamhane’s T2 multiple comparison statistical analyzes were used.

**Results:**

Highest TP values (11.82±0.97) were obtained in ZLS specimens that were 0.4 mm in thickness, cemented on A2 background with light shade resin cement. While, the lowest TP values (9.60±0.55) were calculated in LDS samples that had 0.6 mm thickness, cemented on A3 background with neutral shade resin cement.

**Conclusions:**

Material type, thickness, and background shade affected TP values of specimens. The cement and background shade used might change the final translucency of ZLS and LDS ceramics that had 0.4 and 0.6 mm thickness.

** Key words:**Ceramics, lithium disilicate, spectrophotometry, zirconia, dental materials.

## Introduction

Replication of the natural tooth color and translucency that provides vitality to a restoration is a challenging mission in clinical practice (1). Laminate veneer restorations are minimally invasive approaches with increased light transmission and reflection. However, shade matching with natural tooth and masking the underlying structure color can be a challenge for laminate veneer restorations. Ceramic material’s thickness and type, resin cement and the underlying tooth’s shades contributes the esthetic outcomes of laminate veneers (2,3).

Laminate veneer restorations are commonly produced from glass or hybrid ceramics (4). As, LDS ceramic materials have been using for years (5,6), ZLS ceramics combining high flexural strength and increased esthetic properties has been introduced (7). Although both LDS and ZLS ceramics have translucent crystal and glassy phases (8), smaller silicate crystals in lithium silicate matrix causing higher glass content that makes ZLS more translucenct than conventional LDS (9).

Laminate veneer restorations mostly have 0.3–0.7‑mm thicknesses and can adhesively bond to the enamel with a resin cement (4). The masking ability of veneer restorations may be improved by increasing their thickness (10), however this may need an increased tooth preparation that might lead to decrease the bond strength between dental ceramic and tooth and also endanger the pulpal health (11). Although intra-enamel preparation of laminate veneers, which has 0.5 mm depth, is recommended (12). in most cases, veneers are under prepared (<0.4 mm) or over prepared (>0.6 mm) (12).

In clinical routine, degree of translucency and masking ability of restorations should be arranged by most appropriate ceramic material selection with ideal translucency. In a scenario, a ceramic material having lower translucency (13) and opaque color resin cement with higher masking ability (14) need to be used to cover discolored substrate tooth or core.

Although, the influence of cement and background shade on the final color and translucency of ZLS and LDS (15) ceramics with a range of 0.8-2.4 mm thickness have been previously investigated, the comparison of LDS and specific ZLS brand’s (Celtra Duo) TP values in decreased thickness (0.4 and 0.6 mm) is a controversial issue which has not been evaluated. Present study aimed to evaluate the effect of cement and background shades and ceramic thickness on the translucency of LDS and ZLS materials. The hypothesis was that the material type, thickness, cement and background color would not affect the translucency of ceramics.

## Material and Methods

**Figure 1:**
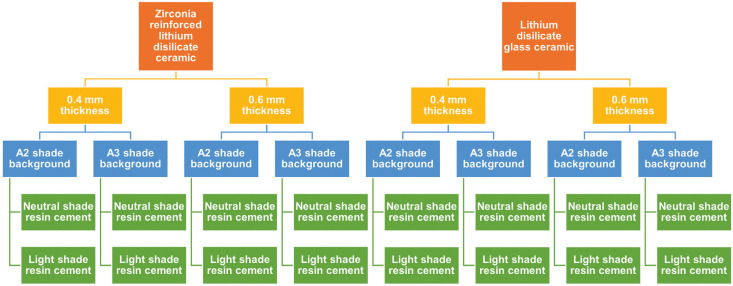
The schematic presentation of study groups.

The required sample size was determined as 10 (n=10) providing a power of at 0.8 and at 0.5 significance, after performing power analysis by a software (G*Power v3.1.9.2; Heinrich-Heine-Universität Düsseldorf). Total sample size was 160 and there were two main groups according to LDS and ZLS materials that were used. Then 16 subgroups were created with respect to thickness (0.4 mm and 0.6 mm), shade of background (A2 and A3), shade of light cured resin cement (light and neutral) (Fig. 1).

**Figure 2:**
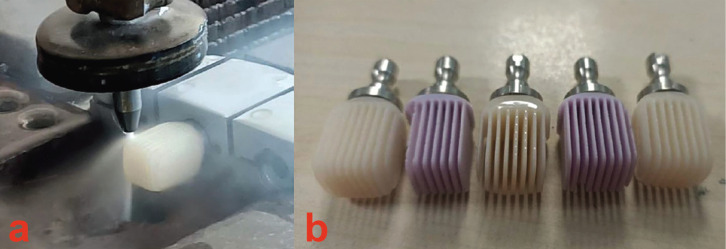
a. Cutting blocks in water jet device, b. View of the blocks after cutting.

A single researcher (O.S.) cut LDS (IPS e.max CAD, Ivoclar Vivadent, Schaan, Liechtenstein) and ZLS (Celtra Duo, Ivoclar Vivadent, Schaan, Liechtenstein) CAD/CAM blocks with 14 mm thickness, A1 color, low translucency (13) with a water jet instrument (DWJ1525-FA; Dardi International Corporation, Nanjing, China) under 25 MPa pressure (Fig. 2). Manufactured specimens’ thicknesses were 0.4 and 0.6 mm (16) Then, according to manufacturer instructions, ceramic specimens entered a furnace (Programat P-310 furnace; Ivoclar Vivadent, Schaan, Liechtenstein) for crystallization process. The thicknesses of each samples were checked by using a digital caliper (Absolute Digimatic Caliper; Mitutoyo Corporation, Aurora, IL, USA).

Nanohybrid composite materials (Estelite Sigma Quick Supra, Tokuyama Dental, Tokyo, Japan) with 5x5 mm dimensions (17) and 2 mm thickness in A2 and A3 shades (18) were used to represent the prepared teeth structure. A metal plate is used to prepare specimens and inner surfaces of the mold were polished for removing the samples easily. After placing composite material in the metal mold without remaining any air bubbles and a transparent glass slab was put on it. Composite was polymerized for 10 s in standard mode (1000 mW/cm2) with the light curing device Valo Grand (Ultradent Products, South Jordan, UT). The thickness and width of the substructure samples were checked by using a caliper.

During surface preparation, the first step was applying 9 % hydrofluoric acid (Porcelain Etch; Ultradent, South Jordan, USA) to the non-glazed surface of LDS and ZLS samples for 20 s, cleaning surfaces with compressed water spray for 20 s and air-drying. Second step was, applying silane (Monobond Plus; IvoclarVivadent, Schaan, Liechtenstein) for 60 s and air-drying. Last step was applying the bonding agent (Adhese Universal; IvoclarVivadent, Schaan, Liechtenstein) for 20 s, and polymerizing it for 10 s in standard mode (1000 mW/cm2) with a light curing device (Valo Grand, Ultradent Products; South Jordan, UT, USA).

**Figure 3:**
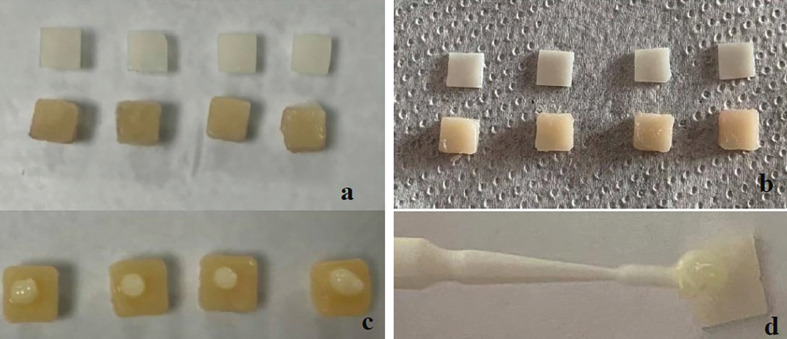
a. Pre-cementation image of ceramic and A3 shade composite background, b. Pre-cementation image of ceramic and A3 shade composite background, c. Placement of resin cement on composite background, d. Placement of resin cement on the surface of ceramic.

Ceramic materials were then cemented to the infrastructure specimens by applying two different shades (neutral, light) light-cure cement (Variolink Esthetic LC, Ivoclar Vivadent, Schaan, Liechtenstein) directly from their tubes. A transparent glass slab was placed and finger pressure was applied over glass for 20 seconds to create a cement layer with 0.1 mm thickness. Following this process, cement was polymerized for 10 s in standard mode by using a light curing device (Valo Grand, Ultradent Products, South Jordan, UT, USA) (Fig. 3).

The the L*a*b* notations of each specimen were measured on black and white backgrounds by using spectrophotometer (VITA EasyShade V, Vita Zahnfabrik, Bad Sackingen, Germany). The translucency parameter (TP), values of each sample were caluculated using this formula; TP=[(Lw* - Lb*)2 + (aw* - ab*)2 + (bw* - bb*)2]1/2. In this formula, subscript ‘b’ represents the color measurements on the black background, and the ‘w’ on the white (19).

A statistical programme (IBM SPSS Statistics, v22.0; IBM Corp Chicago, Illinois, USA) was used to analyse the obtained data. After concluding that the data were normally distributed, the TP values were analysed by descriptive statistics, factorial ANOVA and Tamhane’s T2 multiple comparison tests. The level of significance was determined as α = .05.

## Results

**Figure 4:**
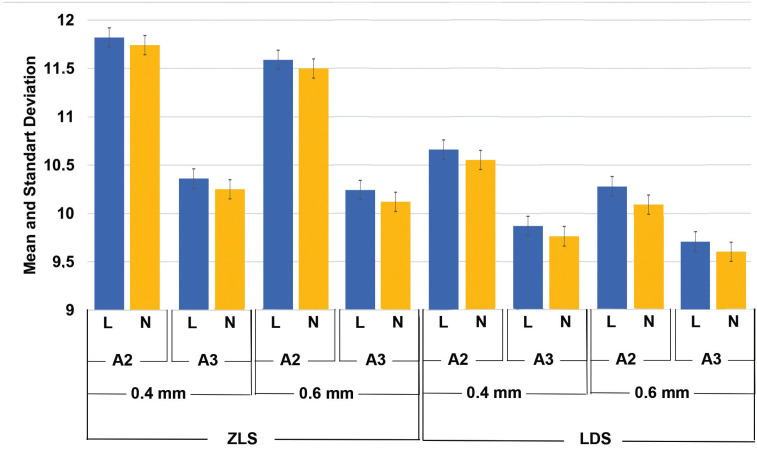
Mean and standart deviation of TP values of different materials, thicknesses background and cement shades.

The specimens’ obtained TP values were different. The highest TP values (11.82±0.97) were obtained in ZLS specimens that were 0.4 mm in thickness, cemented on A2 background with light shade resin cement. While, the lowest TP values (9.60±0.55) were calculated in LDS samples that had 0.6 mm thickness, cemented on A3 background with neutral shade resin cement (Fig. 4).

The factorial ANOVA test results revealed that the type of material affected TP values of specimens (df=1; F=61.39; *P*<.001). Mean TP values of LDS specimens (10.07±0.07) were lower than ZLS samples (10.96±0.12). Also, the effect of material thickness on TP values found to be significant (df=1; F=4.28;P=.040). Mean TP values of samples that had 0.6 mm thickness (10.39±0.22) were lower than specimens that were 0.4 mm in thickness (10.63±0.12). Shade of backgrounds also affected TP values (df=1; F=83.59; *P*<.001). Samples those translucency values measured on A2 shade background were higher (11.03±0.12) than specimens that were measured on A3 shade background (9.99±0.24). Specimens that were cemented with light shade resin cement (10.57±0.16) had higher TP values than samples cemented with neutral shade (10.45±0.34). However cement shade (df=1; F=0.97; *P*=.327) did not significantly affect the TP values of specimens.

## Discussion

The hypothesis that the material type and thickness, background and cement shade would not have impact on the translucency of ceramics was rejected. The ceramic materials’ crystal content’s type, size, and amount can change the degree of translucency that controls the light transmission and reflection thus, directly influence the color masking ability of a veneer (4,20). As it was previously explained, translucency of the ZLS was increased by forming four times smaller silicate crystals (9). In previous studies, a ZLS brand (Celtra Duo) exhibited higher translucency values than conventional LDS (IPS e.max CAD) (9,21) in consistency with the current study.

Restoration thickness influence the translucency of a ceramic material (22). The thinner (0.5 mm) LDS and ZLS materials had a greater translucency values than thicker (0.7 and 1 mm) samples (23). In this study, although the materials’ brands were different, TP values decreased when material thickness increased similarly.

The improved translucency of LDS and ZLS may create a color-matching challenge due to the light that can reflect from the underlying background. The final translucency and color of LDS(20) and ZLS (15) influenced by color of cement and background. As evidence, it has been stated that (24-26), background color affects the final translucency and color of LDS restorations and using opaque shade cement or low translucency ceramic materials to mask dark backgrounds was suggested. Similarly, in this study, although low translusent ZLS and LDS materials were used, background color affected the final translucency. Using translucent and neutral shade resin cement may cause a decreased masking ability in the current study.

Cement color’s effect on the final translucency and color is less important than other parameters when samples have 1.0 to 2.0 mm the thickness. However, a study revealed that, if thickness of a ceramic decreases to 0.5 mm, translucency will improve significantly and can easily be affected by resin cement shade (27). It has been previously stated in a research which were conducted over a light (A1) shaded background and with the LDS and ZLS materials that, opaque cement shade resulted in a higher change in color and translucency than a translucent cement (15). In the present study, unsimilarly, the resin cement shade (translucent and neutral) did not affected the translucency of specimens that had 0.4 mm and 0.6 mm thickness, due to translucent and neutral shades of resin cement may have similar amounts of opacity “ingredients” (28).

The current study contained some limitations. Firstly, aging procedures were not performed and effect of intraoral conditions were not investigated. Secondly, specimens’ color change values of the were not calculated. Thirdly, only one brand ZLS was used. Nevertheless, different brands can display different optical properties. Finally, only two shades of resin cements (clear and neutral) were studied. Various cement shades could give different results. Future researches may study effect of cement thickness and different materials’ shades on the final translucency and color of ceramic materials.

## Conclusions

The TP values of specimens were influenced by material composition and thickness, and background shade. Specimens that were produced from ZLS, 0.4 mm in thickness, cemented on A2 background with light shade resin cement had higher TP values than those manufactured from LDS that had 0.6 mm thickness cemented on A3 background with neutral shade.
